# Retraction: Deregulation of LIMD1-VHL-HIF-1α-VEGF pathway is associated with different stages of cervical cancer

**DOI:** 10.1042/BCJ20170649_RET

**Published:** 2025-06-02

This article (“Deregulation of LIMD1-VHL-HIF-1α-VEGF pathway is associated with different stages of cervical cancer” DOI: 10.1042/BCJ20170649) is being retracted from the *Biochemical Journal* at the request of the Editor-in-Chief and the Editorial Board. This follows similarities being noted by the Editorial Office between figures in two papers from this author group. The first noted is between the Figure 1 HIF-1α #3678 N panel from this paper and the Supplementary Figure S2C p-RB1 x #6261 N panel from Chakraborty et al, 2016 (DOI: 10.1042/BCJ20160323). The second is between this paper’s Figure 1 VHL x #4554 N panel and the Supplementary Figure 3 RBSP3 x #3450 N panel from Chakraborty et al, 2016 (DOI: 10.1042/BCJ20160323).

The authors were contacted regarding the concerns raised and provided an explanation along with the original histology images; however, these have not resolved the issues identified. The Editorial Board therefore stands by the decision to retract.

